# Old Homestead

**Published:** 1889-07

**Authors:** 


THE June number of *The Old Homestead*, published at Savannah, Ga., by Davis Bros., is before us. It contains "Cleopatra," by H. Rider Haggard, and "Bentley's Bride," by Wiegand, both of which are fascinating serials. "Two Women of Shakespeare," by Mamie Neyland, and "The History of the Common People of England," by Julia A. Flisch, are papers that would grace any magazine in Europe or America. "Anna Karenina," a criticism on Tolstoi's novel by Percival S. Menken, and "Cutifachiqui's Treasures," by Meta Telfair McLaws, are contributions that reflect credit on these gifted writers. Palmer's exquisite ode to "Light," "The World from the Sidewalk," and "The Hermitage" furnish the lovers of poetry with choice thoughts. A serial, "Aftermath," by a brilliant young Georgia lady, concludes the literary portion of the magazine. *The Old Homestead* also contains editorials, farm notes, scientific articles, choice miscellaneous matter, puzzle department and several pages of select vocal and instrumental music. It is the only magazine of a literary character in the South and is one of the handsomest publications in the country. A prominent feature of the magazine is the children's page, conducted by Mrs. Harriet A. Sawyer. The July number will be a superb one. The price of this handsome magazine is only fifty cents a year. Sample copy and premium list sent on application. Davis Bros., publishers and proprietors, Savannah, Georgia.

